# Visual Analysis of Uterine Adhesion Research Based on CiteSpace: Bibliometric Analysis From 2006 to 2021

**DOI:** 10.3389/frph.2022.757143

**Published:** 2022-05-09

**Authors:** Dou-Dou Ding, Man-Zhen Zuo, Quan Zhou, Ze-Xian He

**Affiliations:** Department of Obstetrics and Gynecology, The People's Hospital of China Three Gorges University/The First People's Hospital of Yichang, Yichang, China

**Keywords:** intrauterine adhesionis, Ashman's syndrome, CiteSpace, visualization analysis, knowledge mapping

## Abstract

**Background:**

Intrauterine adhesionis caused by a variety of reasons, such as damage of the endometrial basal layer, adhesion or occlusion of the uterine cavity or cervix in different degrees. Seriously endangering women's physical and mental health.

**Objective:**

The purpose of this paper is to analyze the research development of intrauterine adhesions in recent 15 years, explore the future development direction, and promote the development of this field.

**Methods:**

With intrauterine adhesions and Ashman's syndrome as the theme, the related literatures from January 2006 to July 2021 in the Web of Science were searched, and the visual atlas was analyzed by CiteSpace software.

**Results:**

A total of 644 literatures were included. The key words related to intrauterine adhesion mainly include adhesion, pregnancy, expression, intrauterine adhesions, women, adhesion molecule, diagnosis, activation, hysteroscopy and fertility, etc. Six clusters were obtained by keywords analysis, involving hysteroscopy, placenta, office hysteroscopy, uterus and laparoscopy. Co-occurrence of keywords shows that the research focus in recent years is on endometrial repair and regeneration.

**Conclusions:**

Through the bibliometric analysis of WOS research on intrauterine adhesions in recent 15 years, the comprehensive analysis of countries, institutions, authors and keywords is obtained, which has a clear guiding significance for guiding the future development of intrauterine adhesions.

## Introduction

Intrauterine adhesion (IUA) was first proposed by Heinrich Fritsch in 1894 (1), and Joseph G Asher-Man published a series of papers in 1948 to name the disease and describing it in detail, so it is also called Asherman syndrome (2). IUA is a common intrauterine disease, referring to adhesion or even occlusion of uterine isthmus and cervical canal caused by various reasons (3–5). After the occurrence, it will cause serious harm to the reproductive function of patients (6, 7), and IUA patients may face serious obstetric complications (8), including placenta previa, placenta accreta and fetal growth restriction (9–12). In recent years, with the increasing number of intrauterine operations such as induced abortion and curettage, the incidence of intrauterine adhesion has also shown an increasing trend year by year (13, 14). Because of the high incidence and recurrence rate of the disease, the disease is still difficult to treat at present (15). Hysteroscopic adhesiolysis is the most effective method to treat intrauterine adhesion, however the rate of re-adhesion formation after surgery is still high (16). At present, the conventional clinical treatment treatment methods can't solve the serious intrauterine adhesion problem, which can only lead to limited effect and long treatment period (17).

It is essential to understand the research hotspots of intrauterine adhesion for studying effective treatment methods. Understanding the current research trends and hotspots will help medical workers keep up with the development and changes of professional research and provide reference for future professional research and high-quality practical work. Therefore, we use bibliometrics to help us understand the research focus and direction of intrauterine adhesion in the past 15 years.

Bibliometrics is a subject which uses mathematical and statistical methods to quantitatively analyze information (18, 19). It is a comprehensive knowledge system which integrates mathematics, statistics and philology that pays attention to quantification (20, 21). It uses statistical indicators to measure the achievements in this research field (22, 23). It can not only qualitatively and quantitatively analyze the contributions and cooperation of authors, institutions, countries and journals (24, 25), but also make an in-depth assessment of the theme trends and focuses in a certain field (26, 27).

Although bibliometric analysis has been widely used in many disciplines, it is rarely used in the medical field, especially in obstetrics and gynecology. Up to now, there is no comprehensive study on intrauterine adhesions by bibliometrics. This paper aims to use CiteSpace, a free computer program, to analyze the current research progress and future development trend in the field of intrauterine adhesions in recent 15 years from a longitudinal perspective, to help medical workers accurately grasp the research trends and hot spots.

## Materials and Methods

### Research Method

CiteSpace (version 5. 7 R2) is a Java application that realizes the visualization of the retrieval bibliographic database (28). This software can show the development trend and trend of a certain discipline or knowledge field in a certain period, and form the evolution process of several research frontier fields (29, 30). It is vividly presented in front of us in the form of a map (31, 32). Finally, it is analyzed and summarized manually to gain a general grasp of a certain research field.

### Data Source and Retrieval Methods

We searched the Web of Science (WoS, http://login.webofknowledge.com) comprehensively (33). It is a large comprehensive, multidisciplinary and core journal citation index database (34), then this database has been used for bibliometric research before (35–37). Use topic search, and enter “intrauterine adhesion” or “aherman syndrome” in the search box. The search time is from January 1985 to July 2021. The literature sources are academic journals, and to avoid deviation caused by frequent database updates, all searches and data downloads were completed on July 21st, 2021, the results are exported in refworks format, a total of 816 records were retrieved.

### Data Reduction

**Figure 1:**
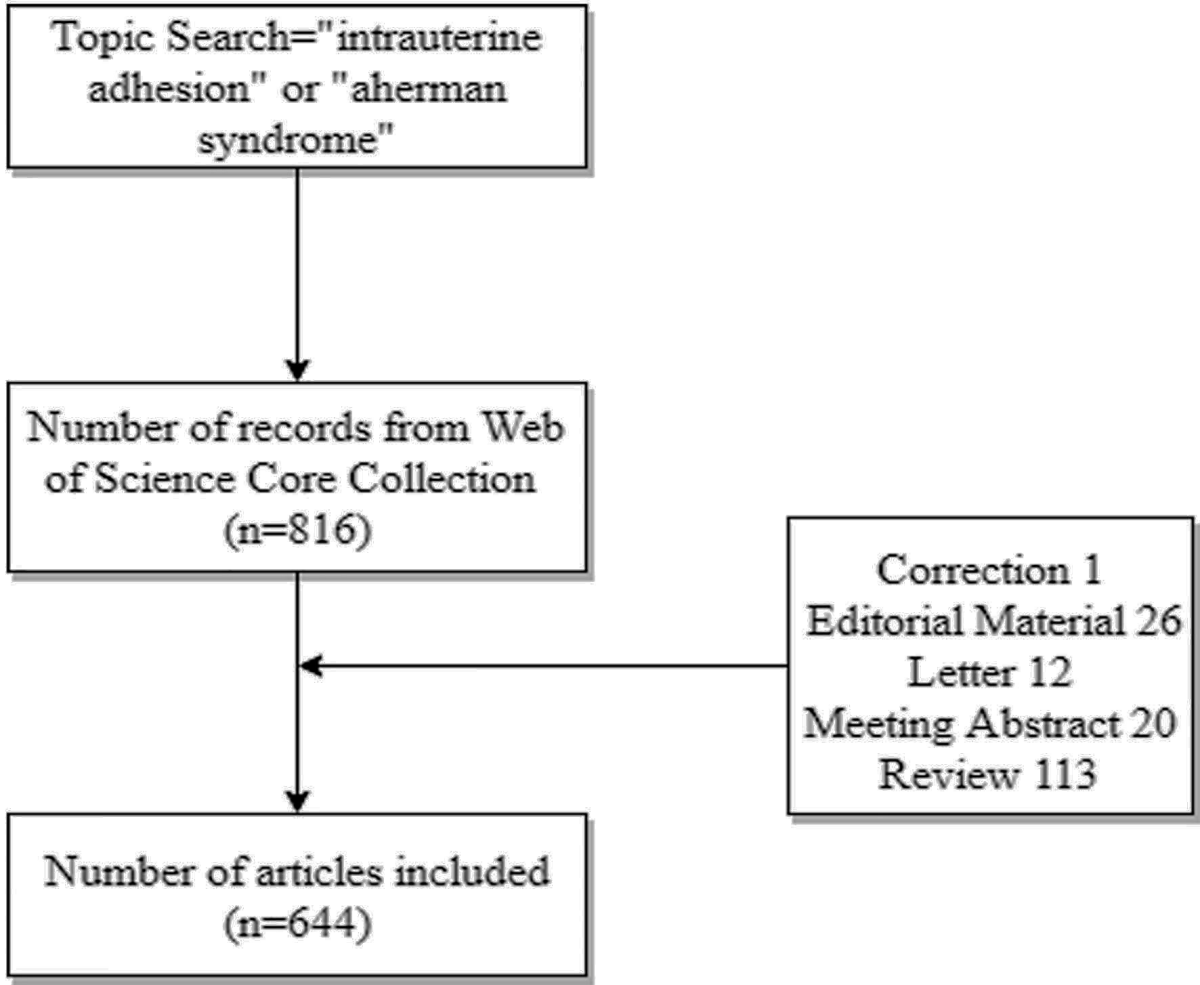
The process of screening articles in bibliometric analysis.

Using CiteSpace, 816 records were scientifically processed, and correction (1), editorial material (26), letter (12), meeting abstract (20) and review (113) were removed, and 644 valid documents were obtained, and the detailed information is shown in Figure 1. The time slicing area is set from 2006 to 2021 to deal with the literature from 2006 to 2021, and node type is checked one at a time, including author, institution, country, keyword.

## Results

### Analysis of Published Articles

**Figure 2:**
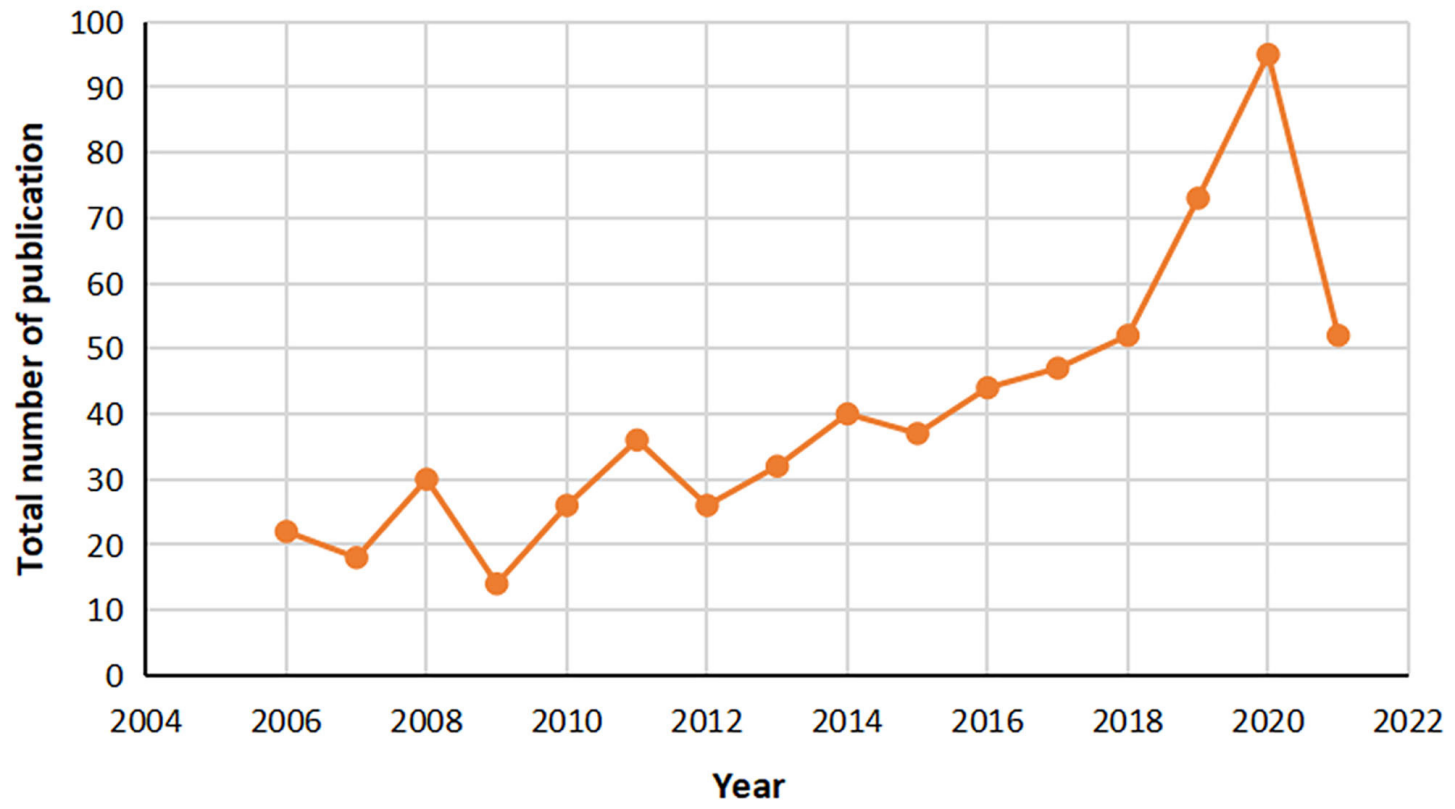
Annual publication curve of WOS (2006–2021) in the field of intrauterine adhesion. The abscissa in the figure represents the year and the ordinate represents the total number of publications.

In the past 15 years from 2006 to 2020, the annual distribution of research papers related to intrauterine adhesion is shown in Figure 2. As can be seen from the figure, since 2006, the number of articles published has been increasing year by year, and the upward trend in 2008 from 2020 is clearly higher than that in previous years.

### Countries and Institutions Analysis

**Figure 3:**
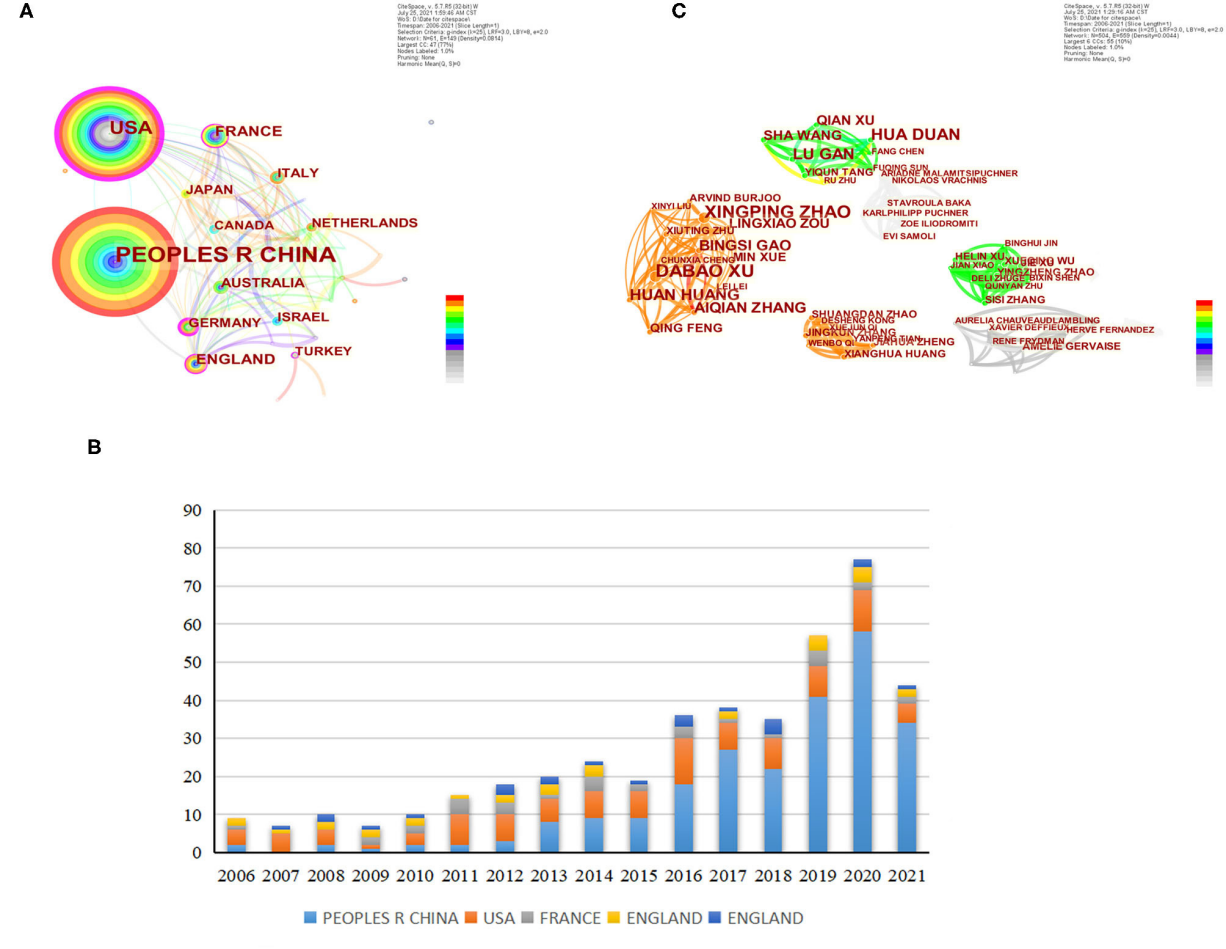
The visual images of countries and institutions generated by CiteSpace software. **(A)** The visual images of countries. **(B)** Annual circulation of the top 5 countries with total circulation from 2006 to 2021. **(C)** The visual images of authors.

During this period, a total of 61 countries have published articles on this research field, and China (Circulation = 238) is the country with the largest contribution, followed by the United States (Circulation = 103), France (Circulation = 32), Britain (Circulation = 30) and Australia (Circulation = 23), in Figures 3A,B and Table 1. And from 2006 to 2021, more than 385 institutions published articles in this field. Capital Med Univ was the institution with the most publications (34), as shown in Table 2.

**Table 1:** Top 10 countries by publishing frequency.

**NO**.	**Countries**	**Year**	**Count**	**Centrality**
1 2 3 4 5 6 7 8 9 10	Peoples R China Usa France England Australia Germany Netherlands Japan Israel Italy	2006 2006 2006 2006 2007 2007 2007 2006 2006 2006	238 103 32 30 23 22 21 20 19 19	0.00 0.48 0.18 0.16 0.04 0.20 0.04 0.00 0.01 0.01

*Year indicates the number of years in which articles were first published, and count indicates the number of articles published during this period*.

**Table 2:** Top 10 institutions by publishing frequency.

**NO**.	**Institutions**	**Year**	**Count**
1 2 3 4 5 6 7 8 9 10	Capital Med Univ Cent South Univ Zhejiang Univ Chinese Univ Hong Kong Southern Med Univ Chongqing Univ Chinese Aced Sci All India Inst Med Sci Tel Aviv Univ Fudan Univ	2012 2020 2016 2014 2016 2008 2006 2007 2014 2014	34 20 15 14 9 9 9 8 7 7

*Year indicates the number of years in which articles were first published, and count indicates the number of articles published during this period*.

### Authors Analysis

As shown in Figure 3C and Table 3, during the period from 2006 to 2021, more than 504 authors published articles, among which DABAO XU was considered the most active author in this field, with the largest number of articles (number = 10), XINGPING ZHAO the second most (number = 9), HUA DUAN (number = 8), HUAN HUANG (number = 7), LU GAN (number = 7), and they are closely related.

**Table 3:** Top 10 authors by publishing frequency.

**NO**.	**Authors**	**Year**	**Count**
1 2 3 4 5 6 7 8 9 10	Dabao Xu Xingping Zhao Hua Duan Lu Gan Huan Huang Aiqian Zhang Songying Zhang Bingsi Gao Xiaona Lin Angelo B Hooker	2015 2020 2016 2016 2020 2015 2018 2020 2018 2015	10 9 8 7 7 6 6 6 6 5

*Year indicates the number of years in which articles were first published, and count indicates the number of articles published during this period*.

### Keyword Analysis

**Figure 4:**
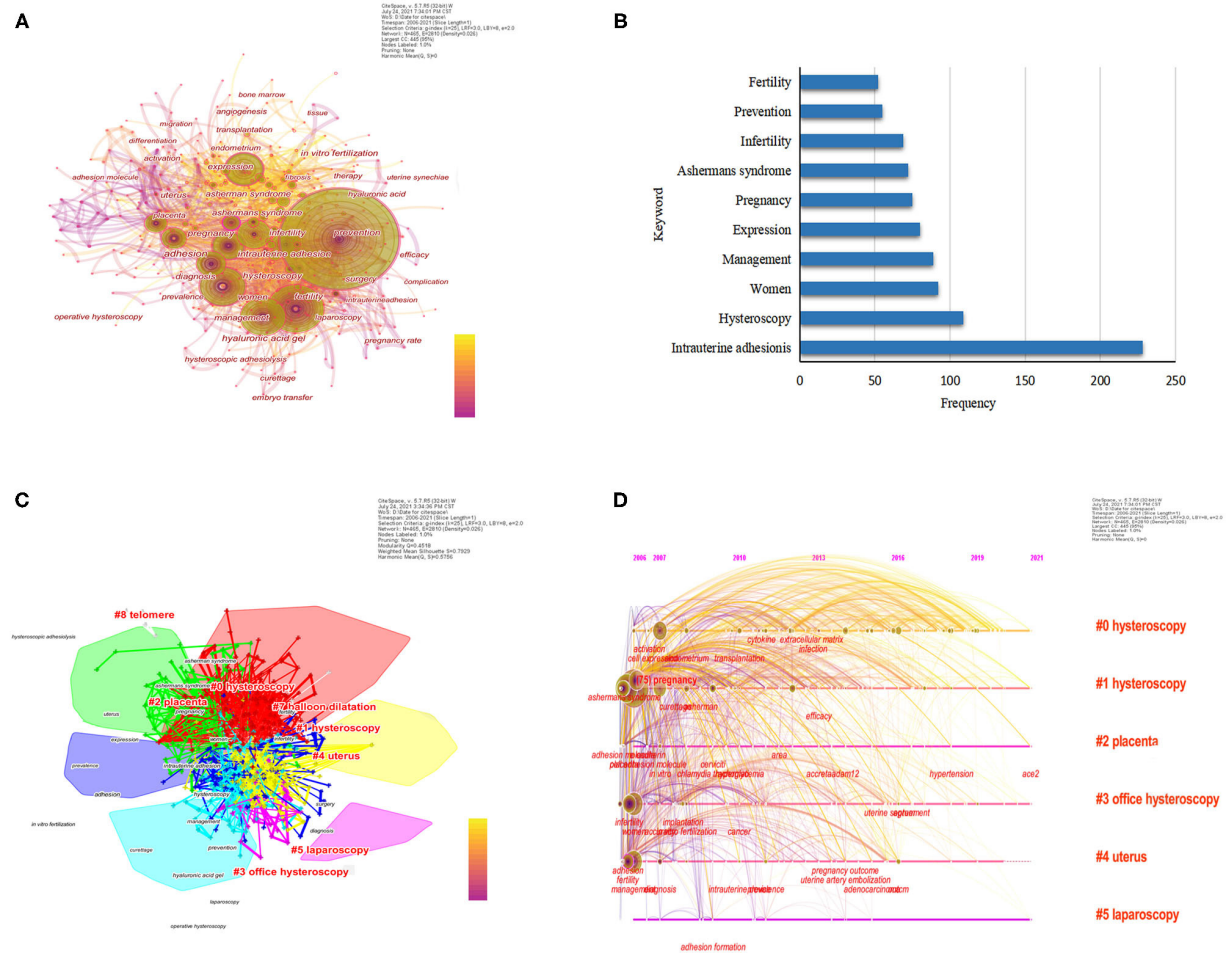
The visual images of keywords generated by CiteSpace software. **(A)** The visual images of the totel of keywords. **(B)** Top 10 keywords with the highest frequency. **(C)** The visual images of cluster analysis of all keywords. **(D)** The timeline zone of top 8 clusters.

**Figure 5:**
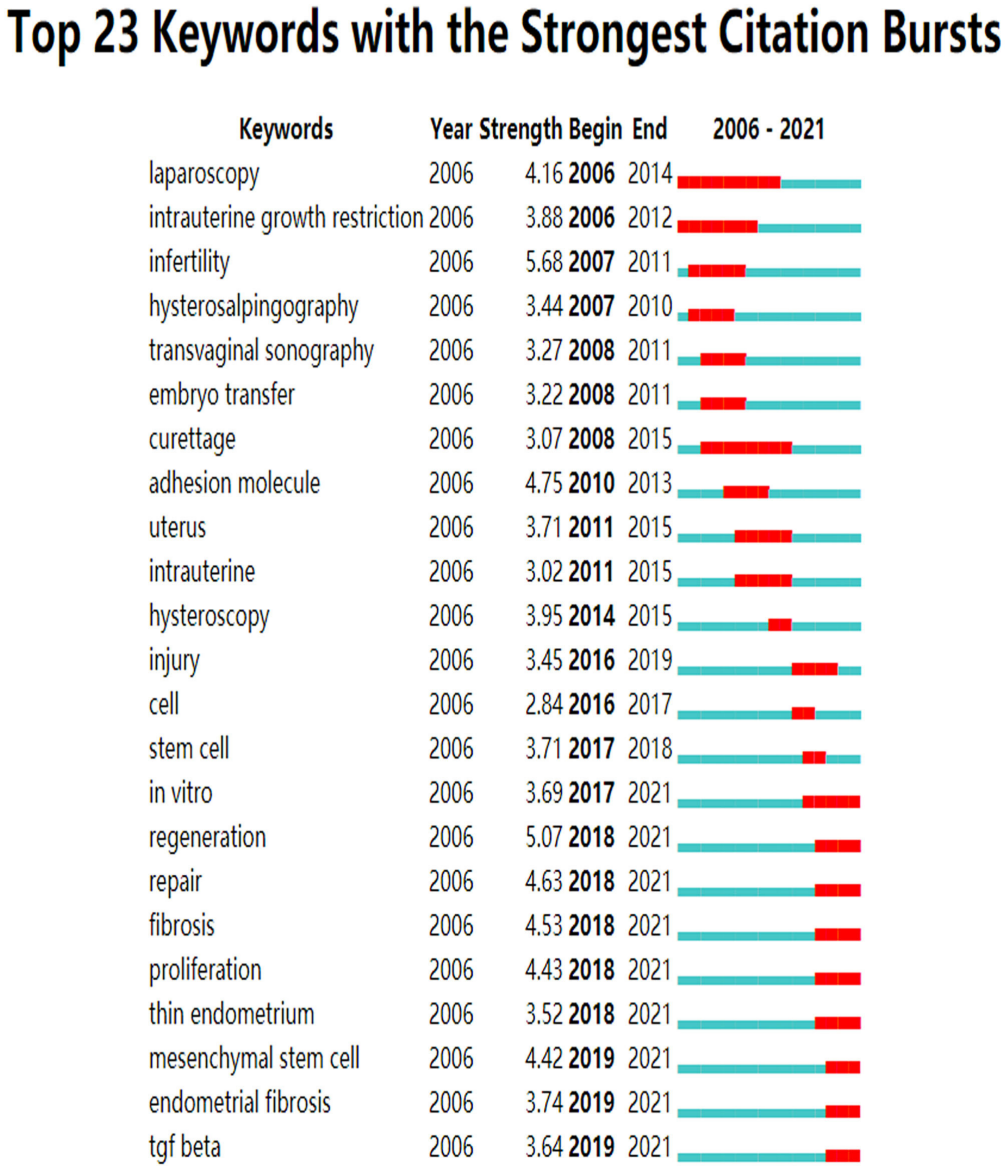
Burst of keywords in CiteSpace. Red indicates the duration of a burst, indicating that keywords are frequently quoted, while green indicates keywords that are not frequently quoted.

A total of 465 keywords were obtained from 614 literatures, and a co-occurrence map of keywords was drawn, as shown in Figure 4A. Among them, the ten most frequently used keywords were intrauterine adhesionis (frequency = 228), hysteroscopy (frequency = 109), women (frequency = 92), management (frequency = 89), expression (frequency = 80), pregnancy (frequency = 75), ashermans syndrome (frequency = 72), infertility (frequency = 68), prevention (frequency = 55) and fertility (frequency = 52), in Figure 4B. And in Table 4, the most significant keywords were adhesion (centrality = 0.24), pregnancy (centrality = 0.20), expression (centrality = 0.13), intrauterine adhesionis (centrality = 0.12), women (centrality = 0.10), adhesion molecule (centrality = 0.09), diagnosis (centrality = 0.08), activation(centrality = 0.08), hysteroscopy (centrality = 0.07) and fertility (centrality = 0.07). Then we obtained the largest 6 clusters as shown in Figures 4C,D and Table 5, and the modularity Q is 0.4518, the weighted mean silhouette S is 0.8038. Summarizing the main research directions of the six clusters, we found that most of them are treatments for intrauterine adhesions. After analyzing the keywords by timeline, it is found that new keywords appear almost every year. As shown in Figure 5, according to keyword burst analysis, during 2006–2021, there were 23 keywords to be found to have the strongest citation outbreak. It can be observed that before 2014, the research hotspots of intrauterine adhesions mainly focused on the treatment of intrauterine adhesions, infertility and assisted reproductive technology, while in recent years, the hotspots focused on stem cell technology, endometrial repair and regeneration. Moreover, the concept of endometrial repair and regeneration which was proposed in 2018 has the highest citation rate, and is considered as the hottest and newest research direction in this field.

**Table 4:** The top 10 keywords sorted by centrality.

**NO**.	**Keywords**	**Centrality**
1 2 3 4 5 6 7 8 9 10	Adhesion Pregnancy Expression Intrauterine adhesionis Women Adhesion molecule Diagnosis Activation Hysteroscopy Fertility	0.24 0.20 0.13 0.12 0.10 0.09 0.08 0.08 0.07 0.07

**Table 5:** The analysis of the top 6 clusters.

**ClusterID**	**Size**	**Silhouette**	**Mean (Citee Year)**	**Label (LLR)**
0 1 2 3 4 5	129 79 75 69 63 22	0.817 0.798 0.842 0.731 0.773 0.867	2016 2012 2008 2011 2011 2010	Hysteroscopy Hysteroscopy Placenta Office hysteroscopy Uterus Laparoscopy

*The cluster analysis results mainly include cluster ID, mean year, size, silhouette, label. Size expresses the number of keywords contained in the cluster, and Mean Year expresses the average year of documents in the cluster*.

## Discussion

In this paper, CiteSpace was used to retrospectively analyze 644 papers on intrauterine adhesion since 2006, and the publication time distribution, cooperation network of the main authors, research hotspots and development trend of the papers in the field of intrauterine adhesion were intuitively reflected by the visual analysis of the knowledge map.

It can be seen from the publishing trend of the article that the published volume is steadily increasing year by year, this shows that intrauterine adhesions are paid increasingly attention by medical workers, and China has made great contributions to the diagnosis and treatment of intrauterine adhesions. And through our analysis, we also find that not only the countries and regions involved in the research are relatively concentrated, but also the research institutions and researchers are relatively concentrated, and the centrality value is not very high.

According to the burst test of keywords, in recent years, we have paid increasingly attention to the repair and regeneration of endometrium, and stem cell therapy technology is also a hot research topic now. This will become the direction of our follow-up research. Based on the existing work, we will deeply explore the pathogenesis and diagnosis and treatment of intrauterine adhesions.

In summary, we have made a bibliometric analysis of the research hotspots and research reversals of intrauterine adhesions. Intrauterine adhesion has been paid close attention to by a wide range of medical workers, and increasingly clinical studies have promoted the diagnosis and treatment of intrauterine adhesion and the invention of new technologies. In the future, more multicenter in-depth research is needed to further provide evidence-based medicine research. This may have obvious guidance for the future work of researchers and medical workers. However, CiteSpace's analysis may be controversial and not deep enough. We will overcome these shortcomings by further evidence-based medicine analysis later.

## Data Availability Statement

The datasets presented in this study can be found in online repositories. The names of the repository/repositories and accession number(s) can be found below: http://login.webofknowledge.com.

## Author Contributions

D-DD designing this study and writing initial draft and revision. D-DD and QZ reviewing the literature and analyzing. Z-XH making figures and tables. M-ZZ rechecking the manuscript and putting forward suggestions for amendment. QZ revising language and content. All authors contributed to the article and approved the submitted version.

## Funding

This study was supported by Hubei Provincial Health Commission Joint Fund Key Project, with Grant No. WJ2019H500.

## Conflict of Interest

The authors declare that the research was conducted in the absence of any commercial or financial relationships that could be construed as a potential conflict of interest.

## Publisher's Note

All claims expressed in this article are solely those of the authors and do not necessarily represent those of their affiliated organizations, or those of the publisher, the editors and the reviewers. Any product that may be evaluated in this article, or claim that may be made by its manufacturer, is not guaranteed or endorsed by the publisher.
